# Meta-analysis Comparing Fluorescence Imaging with Radioisotope and Blue Dye-Guided Sentinel Node Identification for Breast Cancer Surgery

**DOI:** 10.1245/s10434-020-09288-7

**Published:** 2020-11-06

**Authors:** Martha S. Kedrzycki, Maria Leiloglou, Hutan Ashrafian, Natasha Jiwa, Paul T. R. Thiruchelvam, Daniel S. Elson, Daniel R. Leff

**Affiliations:** 1 Hamlyn Centre, Institute of Global Health Innovation, Imperial College London, London, UK; 2 Department of Surgery and Cancer, Imperial College London, London, UK; 3 Department of Breast Surgery, Imperial Healthcare Trust, London, UK

## Abstract

**Introduction:**

Conventional methods for axillary sentinel lymph node biopsy (SLNB) are fraught with complications such as allergic reactions, skin tattooing, radiation, and limitations on infrastructure. A novel technique has been developed for lymphatic mapping utilizing fluorescence imaging. This meta-analysis aims to compare the gold standard blue dye and radioisotope (BD-RI) technique with fluorescence-guided SLNB using indocyanine green (ICG).

**Methods:**

This study was registered with PROSPERO (CRD42019129224). The MEDLINE, EMBASE, Scopus, and Web of Science databases were searched using the Medical Subject Heading (MESH) terms ‘Surgery’ AND ‘Lymph node’ AND ‘Near infrared fluorescence’ AND ‘Indocyanine green’. Studies containing raw data on the sentinel node identification rate in breast cancer surgery were included. A heterogeneity test (using Cochran’s Q) determined the use of fixed- or random-effects models for pooled odds ratios (OR).

**Results:**

Overall, 1748 studies were screened, of which 10 met the inclusion criteria for meta-analysis. ICG was equivalent to radioisotope (RI) at sentinel node identification (OR 2.58, 95% confidence interval [CI] 0.35–19.08, *p *< 0.05) but superior to blue dye (BD) (OR 9.07, 95% CI 6.73–12.23, *p *< 0.05). Furthermore, ICG was superior to the gold standard BD-RI technique (OR 4.22, 95% CI 2.17–8.20, *p *< 0.001).

**Conclusion:**

Fluorescence imaging for axillary sentinel node identification with ICG is equivalent to the single technique using RI, and superior to the dual technique (RI-BD) and single technique with BD. Hospitals using RI and/or BD could consider changing their practice to ICG given the comparable efficacy and improved safety profile, as well as the lesser burden on hospital infrastructure.

**Electronic supplementary material:**

The online version of this article (10.1245/s10434-020-09288-7) contains supplementary material, which is available to authorized users.

The vast majority (~ 90%) of clinically node-negative breast cancer patients undergo a sentinel lymph node biopsy (SLNB) staging procedure.^1^ During SLNB, lymph nodes are sampled to assess whether any metastatic spread has occurred. The aim is to accurately identify the ‘sentinel’ or guardian nodes through lymphatic mapping. This involves injecting a tracer either into or around the tumor, and then subsequently following the drainage pathway to identify the nodes that have ‘taken-up’ the tracer. Conceptually, this is meant to recreate the pathway by which tumor metastasis might spread to axillary nodes. Thus, following this pathway from the tumor to the first lymph node(s) visualizes the sentinel node(s), and the status of these nodes is predictive of the nodal status of the residual axillary basin.^2^^–^5 While there remains a drive to ‘de-escalate’ the surgical management of the axilla to reduce the morbidity associated with axillary lymphadenectomy, it is true that nodal status remains the most powerful determinant of prognosis and subsequent adjuvant therapies.^2^^–^4,6,7

Sentinel node biopsy is an accurate diagnostic technique, and indeed a reliable indicator of the metastatic status of the axilla.^2^^–^4 Gold standard lymphatic mapping uses a combination of blue dye (BD; patent blue, methylene blue, or isosulphan blue) and radioactive colloid.^8^^–^11 This dual tracer approach facilitates high sensitivity and low false negative rates.^9^ However, BD can cause allergic reactions in 1.8% of patients, of which approximately 23% are type I hypersensitivity and 69% are type IV skin reactions.^12^ Additionally, BD can cause semi-permanent skin staining/tattooing, which may or may not fade after several months,^13^ and the technique may fail to accurately identify all sentinel nodes when used in isolation.^14^ Radioactive colloid exposes both the patient and staff to radiation,^15^,^16^ requires constant supply due its short shelf-life (but with limited nuclear reactors capable of making medical grade isotope),^17^ may not be widely available to all hospitals, mandates special licencing as well as hospital infrastructure for safe use and disposal as per the Ionizing Radiation Medical Exposure Regulations,^18^ and when used in isolation fails to give a visual cue to nodal stations. The limitations of both dye and radioisotope (RI) mapping has led to the development of new contrast agents for sentinel node biopsy, such as magnetic^19^,^20^ and fluorescence imaging approaches.^21^^–^31

Fluorescence imaging was first used clinically in the 1940 s, when fluorescein was used in the presence of ultraviolet (UV) light to delineate suspected brain tumors.^32^ Fluorescence imaging with currently approved exogenous fluorophores is a safe, non-ionizing and rapid technique, with macroscopic visualization capabilities to facilitate surgical guidance, as illustrated in Fig. 1. The method combines the use of a fluorescent contrast agent along with specialized cameras designed to capture the fluorescence (in the near-infrared spectral region), as well as visible light emitted from this agent.^33^ A fluorescent contrast agent is a special dye that is able to absorb and then emit light at specific wavelengths.^33^ The imaging system comprises a red/green/blue (RGB) camera that detects light in the visible spectrum, and a monochrome camera sensitive to near-infrared light to detect the light emitted by the fluorophore.^33^ Images are then processed using software that enables the fluorescence image to be overlaid on to the normal visible image,^33^ enabling visualization of the targeted specimen in relation to the surrounding tissue (Fig. 1).

**Fig. 1:**
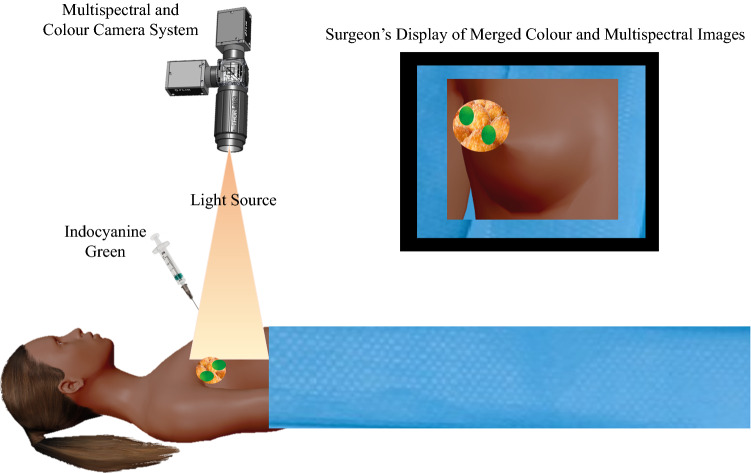
Fluorescence-guided surgery. Indocyanine green is injected periareolarly/intratumorally preoperatively, after which the breast is massaged to distribute the tracer. Intraoperatively, the axillary cavity is exposed to light that activates the fluorescent tracer, and the camera system captures this fluorescence. The signal strength of the lymph nodes is compared with that of the surrounding tissue, and the fluorescence image overlaid on to the color camera image to create a combined picture whereby the lymph nodes are shown to ‘glow green’

Fluorophores, which are European Medicines Agency (EMA)-approved for human use, include indocyanine green (ICG), methylene blue, fluorescein, and aminolevulinic acid (ALA).^34^ Of these, ICG dominates clinical practice, being used for various angiographic studies such as retinography, cardiac function, and liver function.^35^^–^37 ICG has been trialed in various off-label studies for intraoperative analysis of tissue viability during bowel anastomosis or plastic surgery reconstructions, biliary surgery, parathyroid identification, tumor identification, and lymphography and sentinel node identification in various malignancies.^37^^–^40 A number of studies assessing the potential of ICG to aid in sentinel lymph node mapping through the use of fluorescence imaging have now been published, including a critical mass of high-quality prospective studies.^21^^–^30 Previous meta-analyses comparing the sentinel node detection rates between ICG and BD and RI^38^,^41^,^42^ were limited by source data reporting the identification rate per patient (i.e. at least one node was found in each patient) rather than in total nodes, thus obtaining less precise results. The present meta-analysis utilized studies reporting total nodal identification rates for the various modalities, therefore providing a comprehensive comparison on the effectiveness of axillary sentinel node identification using ICG as opposed to the ‘gold standard’ BD and RI.

## Methods

A systematic literature search was performed using the MEDLINE, EMBASE, Scopus, and Web of Science databases for all articles published before September 2019. The study was registered on PROSPERO (CRD42019129224). The following Medical Subject Headings (MeSH) were used in combination and/or with operators: *(*‘*intraoperative*’*[All Fields] OR* ‘*intra*-*operative*’*[All Fields] OR* ‘surger*’*[All Fields] OR* ‘*surgical*’*[All Fields] OR* ‘operat*’*[All Fields] OR* ‘*surgery*’*[MeSH Terms]) AND (*‘sentinel node*’*[All Fields] OR* ‘lymph node*’*[All Fields] OR* ‘axillary node*’*[All Fields] OR* ‘mammary node*’*[All Fields] OR* ‘breast node*’*[All Fields] OR* ‘supraclavicular node*’*[All Fields] OR* ‘*lymph node*’*[MeSH Terms]) AND (*‘*ICG*’*[All Fields] OR* ‘*indocyanine green*’*[All Fields] OR* ‘*indocyanine green*’*[MeSH Terms]) AND (*‘*near infrared*’*[All Fields] OR* ‘*NIR*’*[All Fields] OR* ‘*NIRF*’*[All Fields] OR* ‘multispectr*’*[All Fields] OR* ‘hyperspectr*’*[All Fields] OR* ‘*infrared*’ *[MeSH Terms]).* The search terms were adjusted as needed for each database. Articles were filtered for those focusing on fluorescence imaging of sentinel nodes in breast cancer. Covidence systematic review software^43^ (Veritas Health Innovation, Melbourne, VIC, Australia) was used to deduplicate, screen studies, and extract data through two independent reviewers (MK and NJ). Any conflicting studies were then put to a third reviewer (DRL). Quality assessment was performed using QUADAS2, with only the highest-quality studies. (i.e. those scoring 14/14) being included for meta-analysis.

Studies were only included if the paper (1) focused on sentinel node evaluation in breast cancer; (2) described a clinical trial or cohort study of at least 10 patients; (3) included patients who were undergoing a primary intervention of the breast/axilla; (4) compared ICG with either radiocolloid and/or BD (either patent blue, methylene blue, or isosulphan blue); (5) reported the total number of sentinel nodes identified per tracer; and, finally, (6) full text was available in the English language. Studies were excluded if: (1) the lymph nodes assessed were not related to a primary breast cancer; (2) patients had undergone previous breast and/or axillary surgery and/or received neoadjuvant chemoradiotherapy; (3) ICG was used as the only tracer with no comparator (of BD or RI); (4) they were case studies or studies including < 10 patients; and (5) they were systematic reviews or meta-analyses, animal studies, abstract only, or if the full text was not available in English.

Data extraction included: the type of study, patient number, mean age and range, mean body mass index (BMI) and range, camera used, dyes used, the sentinel node identification rate for each modality, and the metastatic status of the nodes. Statistical analysis was performed using MATLAB (MATLAB and Statistics Toolbox Release 2018b, The MathWorks, Inc., Natick, MA, US).^44^ A heterogeneity test (using Cochran’s Q) determined the use of fixed- or random-effects models for pooled odds ratios (ORs), both of which were reported with 95% confidence intervals.

## Results

**Fig. 2:**
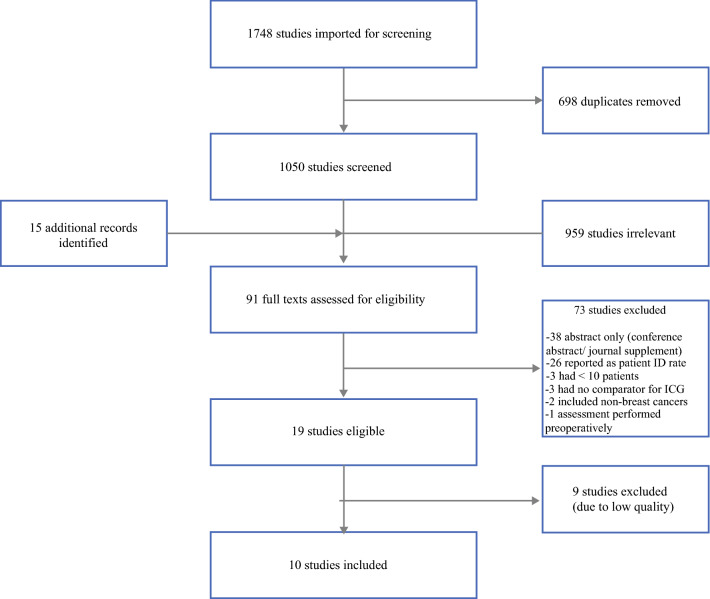
PRISMA flowchart detailing the study selection process. Overall, 1748 studies were identified, of which 698 were duplicates and were subsequently removed. The remaining 1050 studies underwent title and abstract screening, of which 959 were irrelevant. Through cross-referencing, an additional 15 articles were identified and were included in the full-text assessments. Of the full-text articles, only 19 studies met the eligibility criteria, of which only 10 were of high quality. *PRISMA* Preferred Reporting Items for Systematic Reviews and Meta-Analyses

Overall, 1748 articles were identified from the initial literature search. After de-duplication, the remaining 1050 articles underwent title and abstract screening. Of these, only 88 proceeded to full-text evaluation. Finally, 19 studies met the study inclusion criteria, and were subsequently assessed for quality using QUADAS2 (see electronic supplementary material), and the top-scoring articles for each category of ICG versus RI, ICG versus BD, and ICG versus dual technique (scoring 14 out of a possible 14 points) were included for meta-analysis (Fig. 2). Therefore, 10 studies were included in the final meta-analysis (Table 1).

**Table 1:** Studies included in the meta-analysis

References	Year	Method	No. of patients	Mean age, years	Range	Mean BMI	Range	Camera	Dyes
Ballardini et al.^21^	2013	NRCT	134	56^a^	26–80	23	18–40	PDE	ICG and RI
He et al.^22^	2016	NRCT	99	51	31–72	24.2	18.7–38.8	FIRE	ICG and BD
Mieog et al.^23^	2011	NRCT	24	59.5^a^	33–81	25^a^	18–38	Mini-FLARE	ICGHSA and BD and RI
Pitsinis et al.^24^	2015	Cohort	50	48	20–48	NA	NA	PDE	ICG and BD
Polom et al.^25^	2012	Cohort	28	54.4	31–71	25.9^a^	19–38.3	PDE	ICG and RI
			21	58.1	44–83	26^a^	20–34.6	PDE	ICGHSA and RI
Samorani et al.^26^	2015	NRCT	301	59^a^	35–90	NA	NA	PDE	ICG and RI
Somashekhar et al.^27^	2020	NRCT	100	52.3^a^	30–80	NA	NA	Irillic.nm	ICG and RI and BD
Sorrentino et al.^28^	2018	Cohort	71	62.4	51–74	26.1	21.3–30.9	HD laparoscopic system	ICG and RI
Valente et al.^29^	2018	Cohort	92	59^a^	35–81	27.5^a^	17–51	PDE	ICG and RI
van der Vorst et al.^30^	2012	NRCT	12	67^a^	48–71	28^a^	20–47	Mini-FLARE	ICG and RI
			12	54^a^	39–75	23.5^a^	19–34		ICG and RI and BD

*BMI* body mass index, *NRCT* non-randomized controlled trial, *NA* not available, *PDE* photodynamic eye, *FIRE* fluorescence image-guided resection equipment, *Mini FLARE* mini fluorescence-assisted resection and exploration imaging system, *ICG* indocyanine green, *ICGHSA* indocyanine green conjugated to human albumin, *RI* radioisotope, *BD* blue dye

^a^Median value is provided instead of the mean

Only six of these 10 studies were prospective, non-randomized clinical trials, whereas the remaining four were cohort studies. Five studies used the Photodynamic Eye™ (PDE) camera system (Hamamatsu Photonics, Shizuoka, Japan), two employed the mini-FLARE™ camera system (Beth Israel Deaconess Medical Center, Boston, MA, USA), one utilized the Fluorescence Image-guided Resection Equipment (FIRE) system (Key Laboratory of Molecular Imaging of Chinese Academy of Sciences, Beijing, China), one capitalized on the HD Laparoscopic System™ (Karl Storz, Tuttlingen, Germany), and one exploited the Irillic.nm System (Irillic Pvt. Ltd., Bangalore, India).

In total, these studies encompassed 944 patients receiving sentinel node biopsy as part of their standard cancer treatment using fluorescence imaging alongside a comparator tracer, including radioactive colloid and/or BD. Patient demographics (age, BMI, etc.) were comparable between studies, as demonstrated in Table 1. Cancer subtype was comparable between studies, with the majority of patients (74–83%) having invasive ductal carcinoma, 3–17% with ductal carcinoma in situ, 6–10% with invasive lobular carcinoma, and 13% with mixed disease.^22^^–^25,29,30

Three studies compared all SLNB localization modalities (ICG, RI, and BD), six compared ICG and RI alone, and another two compared ICG and BD alone. Of the ICG-RI groups, one further subdivided the cohort based on whether or not albumin had been combined with ICG. Only one study reported the signal/background ratio (SBR) for the lymph nodes, which ranged from 8.3 to 10.3 in signal strength.^30^ However, the majority set a predetermined threshold SBR for what they would consider to be a positive signal (typically 1.1–1.2).^22^,^23^

**Fig. 3:**
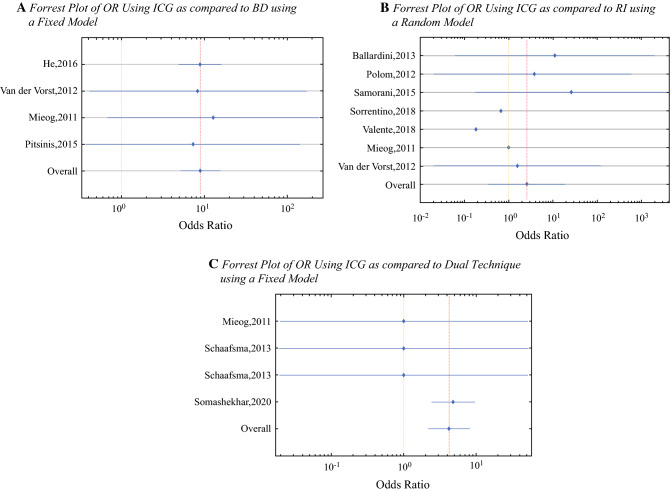
Forrest plots comparing the ORs of identifying a sentinel node using fluorescence imaging compared with the other standard modalities. (**a**) OR using ICG compared with BD using a fixed model. The ORs of identifying a sentinel node using ICG are significantly higher compared with BD (OR 8.89, 95% CI 5.04–15.69). (**b**) OR using ICG compared with RI using a random model. The ORs of identifying a sentinel node using ICG are not significantly different compared with RI (OR 2.58, 95% CI 0.35–19.08). (**c**) OR using ICG compared with the dual technique using a fixed model. The ORs of identifying a sentinel node using ICG are significantly higher compared with the dual technique (OR 4.22, 95% CI 2.17–8.20). *OR* odds ratio, *ICG* indocyanine green, *BD* blue dye, *RI* radioisotope, *CI* confidence interval

Four studies, encompassing 185 patients and 430 lymph nodes, compared ICG and BD (Table 2); 417/430 lymph nodes were identified with ICG, whereas 332/430 lymph nodes were identified with BD. ICG identified an additional 0.33 sentinel lymph nodes (95% CI −0.07 to 0.73, *p *= 0.051) per patient than BD, however this was not statistically significant. Without accounting for study power or variance, the odds of detecting versus not detecting a sentinel node using ICG was 32.1 (417 SLNs detected/13 SLNs not detected), and 3.4 for BD (332 SLNs detected/98 SLNs not detected). Given the homogeneity of the data (Q-value = 0.08, with *p* = 0.99), a fixed model was applied to calculate the OR between the two modalities. In fixed-model analysis (which accounted for study variance through weighting), the OR of detecting versus not detecting SLNs when using ICG compared with BD was 8.89 (95% CI 5.04–15.68) (Fig. 3a). Upon random-effect model analysis, the OR of detecting versus not detecting SLNs using ICG as opposed to BD was 9.45 (95% CI 2.23–40.8). The improvement in sentinel node localization with ICG versus BD was statistically significant (*p *= 0.001) [see the electronic supplementary material].

**Table 2:** Comparison between ICG and BD in the sentinel node identification rate

References	Year	No. of patients	Dyes	SLN identification rate		SLN per patient rate	
				ICG	BD	ICG	RI
He et al.^22^	2016	99	ICG and BD	276/289	202/289	2.79	2.04
Mieog et al.^23^	2011	24	ICGHSA and RI and BD	35/35	30/35	1.46	1.25
Pitsinis et al.^24^	2015	50	ICG and BD	87/87	84/87	1.74	1.68
van der Vorst et al.^30^	2012	12	ICG and RI and BD	19/19	16/19	1.58	1.33

*ICG* indocyanine green, *BD* blue dye, *SLN* sentinel lymph node, *ICGHSA* indocyanine green conjugated to human albumin, *RI* radioisotope

Seven studies, encompassing 693 patients and 1278 lymph nodes, compared ICG and RI (Table 3); 1150/1278 lymph nodes were identified with ICG, whereas 1079/1278 lymph nodes were identified with RI. When ICG was used, 0.01 (95% CI −0.37 to 0.35) more sentinel lymph nodes were identified per patient than when compared with RI, however this was not statistically significant (*p *= 0.48). Without accounting for study power or variance, the odds of detecting versus not detecting a sentinel node using ICG was 9.0 (1150 SLNs detected/128 SLNs not detected), and 5.4 for RI (1079 SLNs detected/199 SLNs not detected). Given the heterogeneity of the data (Q-value = 132.60, with *p* = 0), a random model was applied to calculate the OR between the two modalities. The OR of detecting versus not detecting SLNs using ICG versus RI was 2.58 (95% CI 0.35–19.08) (Fig. 3b). No statistically significant difference was found between the two tracer modalities with respect to sentinel node identification (*p *= 0.18).

**Table 3:** Comparison between ICG and RI in the sentinel node identification rate

References	Year	No. of patients	Dyes	SLN identification rate		SLN per patient rate	
				ICG	RI	ICG	BD
Ballardini et al.^21^	2013	134	ICG and RI	245/246	231/246	1.83	1.72
Mieog et al.^23^	2011	24	ICGHSA and BD and RI	35/35	35/35	1.46	1.46
Polom et al.^25^	2012	59	ICG and RI	68/72	58/72	1.15	0.98
Samorani et al.^26^	2015	301	ICG and RI	583/589	458/589	1.94	1.52
Sorrentino et al.^28^	2018	71	ICG alone/RI alone	76/82	78/82	1.07	1.10
Valente et al.^29^	2018	92	ICG and RI	124/235	202/235	1.35	2.20
van der Vorst et al.^30^	2012	12	ICG and BD and RI	19/19	17/19	1.58	1.42
		12	ICG and RI	18/18	18/18	1.50	1.50

*ICG* indocyanine green, *RI* radioisotope, *SLN* sentinel lymph node, *BD* blue dye, *ICGHSA* indocyanine green conjugated to human albumin

For the ICG versus gold-standard group of combined RI-BD, only two high-quality studies were available for comparison.^23^,^27^ If the cohort was expanded to include all eligible studies regardless of quality scoring, then only one more study could be included.^45^ These studies encompassed 156 patients and 373 lymph nodes (Table 4); 363/373 lymph nodes were identified with ICG, whereas 329/373 lymph nodes were identified with the dual technique. ICG identified an additional 0.218 sentinel lymph nodes per patient (95% CI 0.061–0.375, *p *= 0.003), which was statistically significant. Given the homogeneity of the data (*Q*-value = 1.67, with *p* = 0.64), a fixed model was applied to calculate the OR between the two modalities. In fixed-model analysis (which accounted for within-study precision through weighting), the OR of detecting versus not detecting SLNs when using ICG compared with the dual technique was 4.22 (95% CI 2.17–8.2) (Fig. 3c). The improvement in sentinel node localization with ICG versus the dual technique was statistically significant (*p* < 0.001).

**Table 4:** Comparison between ICG and gold standard in the sentinel node identification rate

References	Year	No. of patients	Dyes	SLN identification rate		SLN per patient rate	
				ICG	RI or BD	ICG	RI
Mieog et al.^23^	2011	24	ICGHSA and BD and RI	35/35	35/35	1.46	1.46
Schaafsma et al.^45^	2013	16	half-dose ICG and RI and BD	21/21	21/21	1.31	1.31
Schaafsma et al.^45^	2013	16	ICG and RI and BD	27/27	27/27	1.69	1.69
Somashekhar et al.^27^	2020	100	ICG and RI and BD	280/290	246/290	2.80	2.40

*ICG* indocyanine green, *SLN* sentinel lymph node, *RI* radioisotope, *BD* blue dye, *ICGHSA* indocyanine green conjugated to human albumin

Multiple studies reported no severe adverse events associated with the dyes.^22^,^24^,^26^,^28^ Only Mieog et al.^23^ reported surgical complications such as wound infection or axillary hematoma, which occurred in 3 of 24 patients. No studies reported long-term complications such as decreased sensation, restricted movement, or lymphedema. Surgical times were equivalent for lumpectomy with SLNB irrespective of which dye was used.^28^,^30^ Cost analysis was also reported to be equivalent between dyes, both per surgery and per patient.^28^

## Discussion

This meta-analysis demonstrates that fluorescence-guided sentinel node mapping with ICG is 8.89 times more likely to identify a sentinel node than BD alone, and 4.22 times more likely than the dual technique. Conversely, there was no statistically significant difference in sentinel node identification rates when comparing ICG with RI alone. These results may have significant implications for centers that are unable to use RI or those that only use BD for SLNB, since detection rates could significantly and safely be improved if they were to change practice to using fluorescence imaging with ICG instead.

The difference between the number of sentinel nodes identified per patient was not statistically significant when compared with individual dyes (0.33 more for ICG vs. BD, with *p *= 0.051; 0.01 less for ICG vs. RI, with *p *= 0.48). However, the difference was statistically significant when ICG was compared with the dual technique (0.218 more for ICG vs. dual, with *p *= 0.03). To date, there are no studies comparing the number of sentinel nodes taken and morbidity rates, although it could be hypothesized that the risk for long-term complications such as lymphedema, sensory deficit, and shoulder function increases with each additional node sampled. Unsurprisingly, there was a significant difference in morbidity rates when comparing SLNB with axillary dissection as per the ALMANAC^5^ and ASOCOG^46^ trials (25% vs. 70%, *p* < 0.001), therefore the number of sentinel nodes sampled evidently affects postoperative complications. However, even with the additional statistically significant 0.218 sentinel nodes per patient, we question whether such a modest increase would have a significant clinical impact.

According to the ALMANAC study, which used the dual technique for SLNB, surgeons require approximately 40 cases to become proficient at this procedure.^47^ This is in keeping with the literature review performed by Sanidas et al., which identified that surgeons in specialist centers were able to reliably perform SLNB after 20–30 cases, but required up to 60 cases in community hospitals.^48^ SLNB with BD has been reported to be more difficult to learn than with RI, as RI is able to give guidance through tissue, whereas BD is only useful upon direct visualization of the node.^48^ To date, no trials have evaluated the learning curve of SLNB with ICG, however as ICG is able to provide real-time lymphatic mapping even prior to incision, it could be hypothesized to be the easiest of the three techniques.^49^ However, injury to the lymphatic vessels may cause ICG to leak and give false positive signals. The surgeons included in the above meta-analysis were already proficient at SLNB using the dual technique, therefore any anecdotal comments on ICG SLNB learning curves were related to the novel equipment rather than the procedure itself. Future work should focus on investigating the learning curve in ICG SLNB to ensure its safe clinical adoption.

ICG and RI are able to provide deep insight into SLNB location, prior to skin incision. BD and RI are static in their feedback, as they only provide signal where sufficient accumulation of the tracer has occurred in sentinel nodes. Conversely, ICG is able to provide dynamic real-time visualization of lymphatic channels as ICG travels from the injection site towards the sentinel node. Surgeons are also therefore able to adjust skin incisions accordingly, to facilitate optimal exposure of sentinel nodes. This requires a lesser degree of navigational technical skill for the surgeon than with RI (with only auditory feedback of the highest concentration points via gamma probe) or BD (where the feedback is after dissection upon direct visualization of the node).^50^ Both ICG and BD are administered subdermally by the surgeon at the start of the operation. This therefore adds approximately 4–6 min to the operative time^51^ to enable travel from the injection site to the sentinel node. However, if the time taken for injection of the tracer is excluded, the procedural times are comparable.^51^ Of note, radiocolloid tends to be injected intratumorally by a radiologist up to 1 day prior to the operation, highlighting both the technical difficulty of injecting this tracer, the need for additional staff for procedure and patient monitoring, increased time burden on the patient, and thus increased hospital pathway burden.^50^

Of note, there were no randomized controlled studies comparing ICG with the dual technique. A possible explanation for this is that ICG is still a relatively novel technique that to date has not yet proven equivalence, therefore the safest option was to use combined techniques so as not to risk missing sentinel nodes. However, with the result of the current analysis, perhaps now there is sufficient evidence to indicate that ICG is not inferior to the dual technique or either technique used in isolation, and therefore a randomized controlled trial to confirm superiority could be justified. Furthermore, this trial could be used to also compare complication rates between dyes, since at present all patients have received multiple dyes,^21^^–^25 therefore any adverse events cannot be attributed to a single agent.

Three studies compared ICG SLNB and axillary node clearance;^52^^–^54 however, only two report sentinel node number as opposed to patient number, therefore there were insufficient data to assess the diagnostic accuracy of ICG in identifying cancerous nodes. In the meta-analysis by Pesek et al.,^55^ which compared nodal identification with SLNB tracers and axillary clearance, the false negative rate was 8.6% for BD, 7.4% for RI, and 5.9% for the dual technique. However, given that in contemporaneous practice, axillary clearance is no longer recommended for diagnostic purposes (as SLNB is equivalent in the efficacy of cancer treatment but with far less morbidity),^5^,^56^ such a comparison is not possible for ICG. Nevertheless, the accuracy of ICG could be assessed by performing a trial whereby patients due to undergo axillary clearance for node-positive disease first undergo an SLNB with ICG.

Multiple studies have focused on comparing various fluorophore concentrations or derivations in terms of efficacy. Mieog et al.^23^ found that there is a wide range of effective doses, as it is only at extremes that there is either too minute a dose to facilitate detection or too large a dose that photoquenching occurs. This finding was further supported in the clinical trial performed by Schaafsma et al.,^45^ when two different particle tracer densities of ICG-Tc were tested, and no significant effect on fluorescence intensity or SBR was observed. Moreover, it was hypothesized that combining albumin and ICG would improve uptake and retention in lymph nodes due to the increased size and polarity of the drug, but this was refuted by both the non-randomized clinical trial by Polom et al.^25^ and the randomized controlled trial by Hutteman and colleagues.^57^ These findings have important implications with regard to cost efficiency, as lower dose/particle density of ICG and the lack of conjugation with albumin would be less costly.

According to the literature, many factors have been suggested to affect fluorescence imaging, such as age, BMI, or hospital setting.^22^,^29^,^58^,^59^ There are conflicting results regarding the effects of age on the identification rate of SLN, with some studies reporting decreased SLN detection for those over 60 years of age,^59^ while other studies report no difference.^22^ A high BMI has been suggested to decrease the identification rate, likely due to the limited penetration of the fluorescence through the extra tissue layers.^58^,^59^ Additionally, hospital setting has been suggested as potentially affecting fluorescence imaging; however, in the study by He et al.,^22^ it was not shown to affect SLN identification rates as long as all surgeons were adequately trained.

At present, none of the contrast agents discussed above are capable of selectively accumulating in metastatic lymph nodes, as they are designed to map sentinel nodes and predict the oncological status of the residual nodal basin. This meta-analysis supports this conclusion, as pooled sensitivity and specificity using a bivariate model found RI to be slightly more accurate than ICG at identifying cancerous nodes during SLNB (AUROC: RI = 0.87, 95% CI 0.84–0.89; ICG = 0.69, 95% CI 0.65–0.73), with both techniques being highly sensitive (ICG = 0.96, RI = 0.96) but not specific (ICG = 0.02, RI = 0.17) [see the electronic supplementary material]. A possible explanation for this is that the size/molecular weight of the tracer molecules may affect their ability to flow through the lymphatic system, which may become altered during cancer metastasis.^59^ RI and BD, which are smaller in size, will be more able to pass, whereas ICG, which is a large peptide chain, may be trapped earlier on.^59^ The study performed by Meric-Bernstam et al.^53^ failed to find any difference between the fluorescence of healthy or cancerous lymph nodes; however, this study was insufficiently powered to confirm statistical significance. Nevertheless, the possibility of being able to identify which lymph nodes are macroscopically cancerous at the outset could limit excessive axillary node sampling and ameliorate the risk of subsequent complications. Microscopic disease is unlikely to provide a sufficient signal for fluorescent detection, however given that microscopic deposits can be treated effectively with radiotherapy,^7^ and modern practice is to not perform lymphadenectomy for micrometastatic disease, the demand for such as a metastatic contrast agent would be to highlight nodes replaced by large macrometastatic deposits.

The key to any technology being embraced within the hospital setting is in it being as effective as the current gold standard, at a low cost. Despite fluorescence-guided surgery requiring additional expenditure, such as a dedicated camera and non-reusable materials, overall, there are cost savings associated with this approach over gold-standard dual mapping with BD and RI. For example, Technitium is approximately five times more expensive than ICG.^51^ These additional costs are due to the additional hospital infrastructure to accommodate radioactive substances, specialist staff required for administration, patient monitoring post-injection, and patient travel, as well as the increased cost of the tracer itself.^51^ Furthermore, given the short activity half-life of RIs, with limited nuclear medicine facilities capable of production and distribution, there is also the risk of inability to procure the tracer. The addition of BD to a combined dye–isotope technique adds further material costs ($15–108),^60^,^61^ and is only really financially competitive when used in isolation as it does not necessitate specific hardware, additional hospital visits, or specialist hospital infrastructure. Therefore, from a cost standpoint alone, ICG is preferable to RI and BD for SLNB.

### Limitations

The current study has a number of important limitations that merit consideration. There were no randomized controlled trials comparing outcomes between the different modalities, as all trials to date were comparative cohort studies. There was heterogeneity between studies with regard to dyes being compared, equipment, and hospital setting. There was diversity between studies in dyes as some compared ICG with BD, others compared ICG with RI, and only three studies compared all three dyes. Furthermore, the dose of ICG administered was variable, however ICG has a wide range of doses at which it is effective. Additionally, some studies combined ICG and albumin, however given that in those studies no difference was found between the ICG versus ICG–albumin cohort, these data were included. Despite variation with regard to which cameras were used, all had been tailored to ICG and its unique absorption and emission spectra (805 nm and 830 nm, respectively).^35^ Each camera was equipped with a light source capable of activating ICG, and an NIR camera capable of detecting the emitted light (PDE detects signals > 820 nm, MiniFLARE > 700 and 800 nm, and FIRE at 700–900 nm).^22^^–^24 With regard to variation of hospital setting, although there was no difference in the multicenter trial performed by He et al.,^22^ it could prove more difficult for district general hospitals to embrace new techniques as they may have decreased exposure. We attempted to account for interstudy variability using the QUADAS2 scoring, and only studies with low bias and high applicability were included.

## Conclusion

Fluorescence-guided axillary sentinel lymph node biopsies provide a safe and effective alternative to BD or radiocolloid. SLNB with ICG is not inferior to the dual technique or RI alone, but is superior to BD. The use of ICG would not only be justified in terms of SLNB efficacy, but would eliminate the risks associated with ionizing radiation, skin tattooing, and hypersensitivity reactions.

## Electronic supplementary material

Below is the link to the electronic supplementary material.

Supplementary material 1 (DOCX 364 kb)

## References

[1] Jauhari Y, Gannon M, Medina J, Cromwell DA, Horgan K, Dodwell D. National Audit of Breast Cancer in Older Patients. Healthcare Quality Improvement Partnership National Clinical Audit and Patient Outcomes Programme. 2018. Available at: https://www.hqip.org.uk/wp-content/uploads/2018/06/ref58_Breast-Cancer_NABCOP-2018-Annual-Report-v1.1_correction.pdf. Accessed 2 Dec 2019.

[2] Cox CE, Bass S, Ku NN, Berman CG, Shons AR, Yeatman T (1998). Sentinel lymphadenectomy: a safe answer to less axillary surgery?. Recent Results Cancer Res..

[3] Doting MHE, Jansen L, Nieweg OE, Piers DA, Tiebosch ATMG, Schraffordt Koops H (2000). Lymphatic mapping with intra-lesional tracer administration in breast cancer patients. Cancer.

[4] Giuliano AE, Haigh PI, Brennan MB, Hansen NM, Kelley MC, Ye W (2000). Prospective observational study of sentinel lymphadenectomy without further axillary dissection in patients with sentinel node-negative breast cancer. J Clin Oncol.

[5] Mansel RE, Fallowfield L, Kissin M, Goyal A, Newcombe RG, Dixon JM (2006). Randomized multicenter trial of sentinel node biopsy versus standard axillary treatment in operable breast cancer: the ALMANAC Trial. J Natl Cancer Inst.

[6] Pepels MJ, de Boer M, Bult P, van Dijck JA, van Deurzen CH, Menke-Pluymers MB (2012). Regional recurrence in breast cancer patients with sentinel node micrometastases and isolated tumor cells. Ann Surg.

[7] Donker M, van Tienhoven G, Straver ME, Meijnen P, van de Velde CJH, Mansel RE (2014). Radiotherapy or surgery of the axilla after a positive sentinel node in breast cancer (EORTC 10981-22023 AMAROS): a randomised, multicentre, open-label, phase 3 non-inferiority trial. Lancet Oncol.

[8] Thevarajah S, Huston TL, Simmons RM (2005). A comparison of the adverse reactions associated with isosulfan blue versus methylene blue dye in sentinel lymph node biopsy for breast cancer. Am J Surg.

[9] Ferrucci M, Franceschini G, Douek M (2018). New techniques for sentinel node biopsy in breast cancer. Transl. Cancer Res..

[10] Giuliano A, Kirgan D, Gunther J, Morton D (1994). Lymphatic mapping and sentinel lymphadenectomy for breast cancer. Ann Surg..

[11] Krag D, Weaver DL, Ashikaga T, Moffat F, Klimberg S, Shriver C (1998). The sentinel node in breast cancer: a multicenter validation study. N Engl J Med.

[12] Montgomery LL, Thorne AC, Van Zee KJ, Fey J, Heerdt AS, Gemignani M (2002). Isosulfan blue dye reactions during sentinel lymph node mapping for breast cancer. Anesth Analg..

[13] Gumus M, Gumus H, Jones SE, Jones PA, Sever AR, Weeks J (2013). How long will I be blue? Prolonged skin staining following sentinel lymph node biopsy using intradermal patent blue dye. Breast Care (Basel).

[14] Peek MC, Kovacs T, Baker R, Hamed H, Kothari A, Douek M (2016). Is blue dye still required during sentinel lymph node biopsy for breast cancer?. Ecancermedical Sci..

[15] Bronskill M (1983). Radiation dose estimates for interstitial radiocolloid lymphoscintigraphy. Semin Nucl Med..

[16] S. L. Stratmann, T. M. McCarty, Kuhn JA. Radiation safety with breast sentinel node biopsy. *Am. J. Surg.* 1999;178(6):454–57. 10.1016/s0002-9610(99)00230-5 10670851

[17] Green C (2012). Technetium-99 m production issues in the United Kingdom. J Med Phys.

[18] Ionising Radiation (Medical Exposure) Regulations (IR(ME)R) | Care Quality Commission. Care Quality Commission. Available at: https://www.cqc.org.uk/guidance-providers/ionising-radiation/ionising-radiation-medical-exposure-regulations-irmer. Accessed 2 Dec 2019.

[19] Alvarado MD, Mittendorf EA, Teshome M, Thompson AM, Bold RJ, Gittleman MA (2019). SentimagIC: a non-inferiority trial comparing superparamagnetic iron oxide versus technetium-99 m and blue dye in the detection of axillary sentinel nodes in patients with early-stage breast cancer. Ann Surg Oncol.

[20] Douek M, Klaase J, Monneypenny I, Kothari A, Zechmeister K, Brown D (2014). Sentinel node biopsy using a magnetic tracer versus standard technique: the SentiMAG multicentre trial. Ann Surg Oncol.

[21] Ballardini B, Santoro L, Sangalli C, Gentilini O, Renne G, Lissidini G (2013). The indocyanine green method is equivalent to the (9)(9)mTc-labeled radiotracer method for identifying the sentinel node in breast cancer: a concordance and validation study. Eur J Surg Oncol.

[22] He K, Chi C, Kou D, Huang W, Wu J, Wang Y (2016). Comparison between the indocyanine green fluorescence and blue dye methods for sentinel lymph node biopsy using novel fluorescence image-guided resection equipment in different types of hospitals. Transl Res.

[23] Mieog JS, Troyan SL, Hutteman M, Donohoe KJ, van der Vorst JR, Stockdale A (2011). Toward optimization of imaging system and lymphatic tracer for near-infrared fluorescent sentinel lymph node mapping in breast cancer. Ann Surg Oncol.

[24] Pitsinis V, Provenzano E, Kaklamanis L, Wishart GC, Benson JR (2015). Indocyanine green fluorescence mapping for sentinel lymph node biopsy in early breast cancer. Surg Oncol.

[25] Polom K, Murawa D, Nowaczyk P, Rho YS, Murawa P (2012). Breast cancer sentinel lymph node mapping using near infrared guided indocyanine green and indocyanine green human serum albumin in comparison with gamma emitting radioactive colloid tracer. Eur J Surg Oncol.

[26] Samorani D, Fogacci T, Panzini I, Frisoni G, Accardi FG, Ricci M (2015). The use of indocyanine green to detect sentinel nodes in breast cancer: a prospective study. Eur J Surg Oncol.

[27] Somashekhar SP, Kumar CR, Ashwin KR, Zaveri SS, Jampani A, Ramya Y (2020). Can low-cost indo cyanine green florescence technique for sentinel lymph node biopsy replace dual dye (radio-colloid and blue dye) technique in early breast cancer: a prospective two-arm comparative study. Clin Breast Cancer..

[28] Sorrentino L, Sartani A, Pietropaolo G, Bossi D, Mazzucchelli S, Truffi M (2018). A novel indocyanine green fluorescence-guided video-assisted technique for sentinel node biopsy in breast cancer. World Journal of Surgery..

[29] Valente SA, Al-Hilli Z, Radford DM, Yanda C, Tu C, Grobmyer SR (2019). Near infrared fluorescent lymph node mapping with indocyanine green in breast cancer patients: a prospective trial. J Am Coll Surg.

[30] van der Vorst JR, Schaafsma BE, Verbeek FP, Hutteman M, Mieog JS, Lowik CW (2012). Randomized comparison of near-infrared fluorescence imaging using indocyanine green and 99(m) technetium with or without patent blue for the sentinel lymph node procedure in breast cancer patients. Ann Surg Oncol.

[31] Ahmed M, Purushotham A, Douek M (2014). Novel techniques for sentinel lymph node biopsy in breast cancer: a systematic review. Lancet Oncol.

[32] Moore G, Peyton WT, French LA, Walkter WW (1948). The clinical use of fluorescein in neurosurgery: the localization of brain tumors. J Neurosurg.

[33] Zheng Y, Yang H, Wang H, Kang K, Zhang W, Ma G (2019). Fluorescence-guided surgery in cancer treatment: current status and future perspectives. Ann Transl Med.

[34] Medicines. European Medicines Agency. 2020. Available at: https://www.ema.europa.eu/en/medicines. Accessed 17 Feb 2020.

[35] Marshall MV, Rasmussen JC, Tan IC, Aldrich MB, Adams KE, Wang X (2010). Near-infrared fluorescence imaging in humans with indocyanine green: a review and update. Open Surg Oncol J.

[36] Nagaya T, Nakamura YA, Choyke PL, Kobayashi H (2017). Fluorescence-guided surgery. Front Oncol.

[37] Alander JT, Kaartinen I, Laakso A, Patila T, Spillmann T, Tuchin VV (2012). A review of indocyanine green fluorescent imaging in surgery. Int J Biomed Imaging.

[38] Schaafsma BE, Mieog JSD, Hutteman M, Van der Vorst JR, Kuppen PJK, Lowik C (2011). The clinical use of indocyanine green as a near-infrared fluorescent contrast agent for image-guided oncologic surgery. J Surg Oncol.

[39] van Manen L, Handgraaf HJM, Diana M, Dijkstra J, Ishizawa T, Vahrmeijer AL (2018). A practical guide for the use of indocyanine green and methylene blue in fluorescence-guided abdominal surgery. J Surg Oncol.

[40] Demarchi MS, Karenovics W, Bedat B, Triponez F (2020). Intraoperative autofluorescence and indocyanine green angiography for the detection and preservation of parathyroid glands. J Clin Med.

[41] Te Velde EA, Veerman T, Subramaniam V, Ruers T (2010). The use of fluorescent dyes and probes in surgical oncology. Eur J Surg Oncol.

[42] Zhang X, Li Y, Zhou Y, Mao F, Lin Y, Guan J (2016). Diagnostic performance of indocyanine green-guided sentinel lymph node biopsy in breast cancer: a meta-analysis. PLoS ONE.

[43] *Covidence systematic review software* [computer program]. Melbourne, VIC: Veritas Health Innovation.

[44] MATLAB and Statistics Toolbox Release (2019). computer program.

[45] Schaafsma BE, Verbeek FPR, Rietbergen DDD, Van Der Hiel B, Van Der Vorst JR, Liefers GJ (2013). Clinical trial of combined radio- and fluorescence-guided sentinel lymph node biopsy in breast cancer. Br J Surg.

[46] Giuliano AE, Hunt KK, Ballman KV (2011). Axillary dissection vs no axillary dissection in women with invasive breast cancer and sentinel node metastasis: a randomized clinical trial. JAMA.

[47] Clarke D, Newcombe RG, Mansel RE (2004). The learning curve in sentinel node biopsy: the ALMANAC experience. Ann. Surg. Oncol.

[48] Sanidas EE, de Bree E, Tsiftsis DD (2003). How many cases are enough for accreditation in sentinel lymph node biopsy in breast cancer?. Am. J. Surg..

[49] Papathemelis T, Jablonski E, Scharl A, Hauzenberger T, Gerken M, Klinkhammer-Schalke M (2018). Sentinel lymph node biopsy in breast cancer patients by means of indocyanine green using the Karl Storz VITOM(R) fluorescence camera. Biomed Res Int..

[50] Diaz de la Noval B (2016). Pros and Cons of Radio-Iodine-125, Technetium-99M nanocolloid, indocyanine green fluorescence and new techniques as tumoral tracers and lymphatic mapping in breast conservative surgery. Med. Sci..

[51] Cattin F, Fogacci T, Frisoni G, Fabiocchi L, Dellachiesa L, Semprini G, et al. ICG versus 99tc in breast surgery-how to match quality health care and costs reduction: a cost effectiveness study. *J Cancer Sci Ther* 2017;09(02).

[52] Chi C, Ye J, Ding H, He D, Huang W, Zhang GJ (2013). Use of indocyanine green for detecting the sentinel lymph node in breast cancer patients: from preclinical evaluation to clinical validation. PLoS ONE.

[53] Meric-Bernstam F, Rasmussen JC, Krishnamurthy S, Tan IC, Zhu B, Wagner JL (2014). Toward nodal staging of axillary lymph node basins through intradermal administration of fluorescent imaging agents. Biomed. Opt. Express.

[54] Somashekhar SP, Rohit Kumar C, Ashwin KR, Rakshith S, Jampani A, Ramya Y (2019). Indocyanine Green (ICG) fluorescence imaging in sentinel lymph node biopsy (SLNB) for early breast cancer: first Indian experience. Indian J Gynecol Oncol..

[55] Pesek S, Ashikaga T, Krag LE, Krag D (2012). The false-negative rate of sentinel node biopsy in patients with breast cancer: a meta-analysis. World J Surg.

[56] Kuru B (2020). The adventure of axillary treatment in early stage breast cancer. Eur J Breast Health..

[57] Hutteman M, Mieog JSD, van der Vorst JR, Liefers GJ, Putter H, Lowik C (2011). Randomized, double-blind comparison of indocyanine green with or without albumin premixing for near-infrared fluorescence imaging of sentinel lymph nodes in breast cancer patients. Breast Cancer Res Treat.

[58] Grischke EM, Rohm C, Hahn M, Helms G, Brucker S, Wallwiener D (2015). ICG fluorescence technique for the detection of sentinel lymph nodes in breast cancer: results of a prospective open-label clinical trial. Geburtshilfe und Frauenheilkunde.

[59] Takemoto N, Koyanagi A, Yasuda M, Yamamoto H (2018). Comparison of the indocyanine green dye method versus the combined method of indigo carmine blue dye with indocyanine green fluorescence imaging for sentinel lymph node biopsy in breast conservative therapy for stage ≤ iIA breast cancer. BMC Women’s Health.

[60] Drugs.com. Methylene blue prices, coupons & patient assistance programs. 2020. Available at: https://www.drugs.com/price-guide/methylene-blue. Accessed 17 Sep 2020.

[61] Gold HT, Do HT, Osborne MP (2005). Cost-effectiveness of isosulfan blue vs. methylene blue dye in sentinel node biopsy. J Clin Oncol.

