# Supplementary material for: Meta-analysis Comparing Fluorescence Imaging with Radioisotope and Blue Dye-Guided Sentinel Node Identification for Breast Cancer Surgery
Source: Ann Surg Oncol. 2020 Nov 6;28(7):3738–48. doi: 10.1245/s10434-020-09288-7 (PMC8184731; doi:10.1245/s10434-020-09288-7)
Supplement: Supplementary file 1 — Supplementary material 1 (DOCX 364 kb) [file 10434_2020_9288_MOESM1_ESM.docx]

**Supplementary Material:**

**QUADAS2 Scoring for Quality**

|  |  | Patient Selection | Index Test | Reference Standard | Flow & Timing | Patient Selection | Index Test | Reference Standard |
| --- | --- | --- | --- | --- | --- | --- | --- | --- |
| Ballardini | 2013 | 2 | 2 | 2 | 2 | 2 | 2 | 2 |
| Grischke | 2015 | 2 | 2 | 2 | 0 | 2 | 2 | 2 |
| He | 2016 | 2 | 2 | 2 | 2 | 2 | 2 | 2 |
| Liu | 2017 | 0 | 2 | 2 | 2 | 2 | 2 | 2 |
| Mieog | 2011 | 2 | 2 | 2 | 2 | 2 | 2 | 2 |
| Papathemelis | 2018 | 0 | 2 | 2 | 2 | 2 | 2 | 2 |
| Pitsinis | 2015 | 2 | 2 | 2 | 2 | 2 | 2 | 2 |
| Polom | 2012 | 2 | 2 | 2 | 2 | 2 | 2 | 2 |
| Samorani | 2015 | 2 | 2 | 2 | 2 | 2 | 2 | 2 |
| Schaafsma | 2013 | 2 | 1 | 1 | 0 | 2 | 2 | 2 |
| Sevick-Muraca | 2008 | 1 | 1 | 1 | 2 | 2 | 2 | 2 |
| Someshakhar | 2020 | 2 | 2 | 2 | 2 | 2 | 2 | 2 |
| Sorrentino | 2018 | 2 | 2 | 2 | 2 | 2 | 2 | 2 |
| Sugie | 2013 | 2 | 2 | 2 | 0 | 2 | 2 | 2 |
| Sugie | 2016 | 2 | 2 | 2 | 0 | 2 | 2 | 2 |
| Valente | 2018 | 2 | 2 | 2 | 2 | 2 | 2 | 2 |
| Van der Vorst | 2012 | 2 | 2 | 2 | 2 | 2 | 2 | 2 |
| Verbeek | 2014 | 2 | 1 | 1 | 2 | 2 | 2 | 2 |
| Wishart | 2012 | 2 | 1 | 1 | 2 | 2 | 2 | 2 |

2= high quality, 1= medium quality, 0= low quality

Only studies scoring 14/14 (highest quality studies) were included in the final metanalysis.

**Odds Ratio Calculation for ICG versus Blue Dye**

**Table 1: Analysis of Sentinel Node Detection when using ICG and BD**

|  | **ICG analysis** | | | | |
| --- | --- | --- | --- | --- | --- |
| **Author** | **SLNs detected** | **SLNs not detected** | **Sum SLNs** | **Detected %** | **Not Detected %** |
| He, 2016^22^ | 276.5 | 13.5 | 290 | 0.953448276 | 0.046551724 |
| Mieog, 2011^23^ | 35.5 | 0.5 | 36 | 0.986111111 | 0.013888889 |
| Pitsinis, 2015^24^ | 87.5 | 0.5 | 88 | 0.994318182 | 0.005681818 |
| Van der Vorst, 2012^29^ | 19.5 | 0.5 | 20 | 0.975 | 0.025 |
|  | **Mean** |  |  | **0.977219392** | **0.022780608** |

|  | **BD Analysis** | | | | |
| --- | --- | --- | --- | --- | --- |
| **Author** | **SLNs Detected** | **SLNs Not Detected** | **Sum SLNs** | **Detected %** | **Not Detected %** |
| He, 2016^22^ | 202.5 | 87.5 | 290 | 0.698275862 | 0.301724138 |
| Mieog, 2011^23^ | 30.5 | 5.5 | 36 | 0.847222222 | 0.152777778 |
| Pitsinis, 2015^24^ | 84.5 | 3.5 | 88 | 0.960227273 | 0.039772727 |
| Van der Vorst, 2012^29^ | 16.5 | 3.5 | 20 | 0.825 | 0.175 |
|  | **Mean** |  |  | **0.832681339** | **0.167318661** |

Where ICG= indocyanine green, BD= blue dye, SLN= sentinel lymph node.

Using the data from Table 1, one can calculate a simplified odds ratio:

Odds of detecting SLN with ICG Odds of detecting SLN with BD

= %SLN detected/ %SLN not detected = %SLN detected/ %SLN not detected

= 0.977/0.023 =0.833/0.167

= 42.478 =4.988

$$Odds Ratio (of ICG:BD)= \frac{odds of detecting SLNs with ICG}{odds of detecting SLNs with BD}$$

= 42.478/4.988

= 8.516

However, the odds ratio (OR) reported in this paper also takes into consideration the weight (within study precision) of each study. As the null hypothesis (the OR for the different studies are drawn from a single distribution) cannot be rejected when heterogeneity tested via Chi-squared distribution, the fixed model is used. In this model, instead of calculating the mean of the OR for all cases, the OR is weighted depending on the variance of each study (as demonstrated in Table 2). Woolf’s formula was used to determine standard error and thus variance (rather than the traditional variance calculation) given the sparse data, where for each study:

Variance= (1/(ICG detected+.5)) + (1/(ICG not detected+.5)) + (1/(BD detected+.5)) + (1/(BD not detected +.5))

The weight (inverse of variance) is then multiplied by the natural logarithm OR for each study. Then, by adding the weighted natural logarithm OR for each study (26.058) and diving by the total weight for all studies (11.928), you get the logarithm OR (2.185). When the logarithm OR value is inversed, you get the fixed OR (8.88).

| **Author** | **ICG** | | **BD** | | **OR** | **Variance** | **Weight**  **(1/Variance)** | **Natural Logarithm OR** | **Natural Logarithm OR*Weight** |
| --- | --- | --- | --- | --- | --- | --- | --- | --- | --- |
|  | detected | not detected | detected | not detected |  |  |  |  |  |
| He, 2016^22^ | 276 | 13 | 202 | 87 | 8.85002286 | 0.094058 | 10.63179 | 2.18042 | 23.18176434 |
| Mieog, 2011^23^ | 35 | 0 | 30 | 5 | 12.8032787 | 2.242774 | 0.445876 | 2.5497013 | 1.136851592 |
| Pitsinis, 2015^24^ | 87 | 0 | 84 | 3 | 7.24852071 | 2.308977 | 0.433092 | 1.9807974 | 0.857867903 |
| Van der Vorst, 2012^29^ | 19 | 0 | 16 | 3 | 8.27272727 | 2.397602 | 0.417083 | 2.1129642 | 0.881282166 |
| **Total** |  |  |  |  |  |  | **11.92784** |  | **26.057766** |

**Table 2: Fixed Model Odds Ratio for ICG:BD Sentinel Node Detection**

Where ICG= indocyanine green, BD= blue dye, OR= odds ratio.

**Sensitivity, Specificity, and Accuracy of ICG versus RI sentinel node mapping**

**Figure 1a: Sensitivity and Specificity of ICG as compared to other sentinel node mapping techniques**

**Figure 1b: Sensitivity and Specificity of RI as compared to other sentinel node mapping techniques**

Forrest plots comparing the odds of identifying a cancerous sentinel node using fluorescence imaging (Figure 1a) as compared to radioisotope (Figure 1b). Both ICG and RI are highly sensitive towards mapping sentinel nodes which have cancer (ICG=0.96 [CI 0.91-0.98], RI=0.96 [CI 0.82-0.99]). However, neither method is specific (ICG=0.02 [CI 0.01-0.05], RI=0.17 [0.02-0.63]).

**Figure 2a: Accuracy of ICG at detecting cancerous nodes during sentinel node biopsy**

**Figure 2b: Accuracy of RI at detecting cancerous nodes during sentinel node biopsy**

AUROC curves demonstrating the accuracy at detecting cancerous sentinel nodes using fluorescence imaging as compared to radioisotope. Overall, RI outperformed ICG in terms of accuracy at identifying which nodes were cancerous (AUROC: ICG=0.69 [CI 0.65-0.73], RI= 0.87[CI 0.84-0.89]).
